# Who Said What? The Effects of Cognitive Load on Source Monitoring and Memory for Multiple witnesses' Accounts

**DOI:** 10.1002/acp.70011

**Published:** 2024-11-27

**Authors:** Pamela Hanway, Lucy Akehurst, Zarah Vernham, Lorraine Hope

**Affiliations:** ^1^ School of Psychology, Sport and Health Sciences University of Portsmouth Portsmouth UK

**Keywords:** cognitive load, investigative interviewing, source monitoring, witness memory, working memory

## Abstract

Investigative interviewers are often required to accurately remember information that has been provided by different people. This can be at the scene of an event or during follow‐up investigations in interview rooms. Interviewers must accurately monitor the source of information to differentiate between witnesses' accounts and to assess what information is novel and what has been corroborated by others or by physical evidence. The current research examined the effects of cognitive load on memory and source monitoring accuracy for information provided by multiple witnesses. Participants, under conditions of high cognitive load (HCL) where load was induced via interviewer‐relevant tasks (e.g., formulating questions) or no cognitive load (NCL), watched five mock‐witnesses' accounts of the same crime. Each witness provided several details of the crime that were unique to their individual account. When asked about account details, and which witness had provided each detail, mock‐interviewers' memory accuracy was lower in the HCL condition than the NCL condition. There was no difference between cognitive load conditions for source monitoring accuracy, which was poor regardless of condition.

Investigative interviewers are often required to accurately remember information that has been provided by different people so they can ask appropriate questions, make decisions and pursue further enquiries (College of Policing 2019). The interviews may take place at the scene of an event or during follow‐up investigations in interview rooms. They are cognitively demanding tasks, particularly when executed in conjunction with the other demands of interviewing, such as building a rapport with the witness, paying attention to the witness's needs, and seeking clarification of the information provided (Fisher et al. 2014; Hanway and Akehurst 2018). The current research examined the effects of increasing cognitive demands during a mock‐interviewing task on participants' perceived cognitive load, the accuracy of their memory for detailed information provided by witnesses, and their accuracy for monitoring the source of information provided by multiple witnesses.

Information provided by witnesses during their interviews often forms the basis of an investigation. The accuracy and completeness of witness information can determine the outcome of an investigation. Inaccurate representation of a witness's account by interviewers, for example when recapping evidence either during an interview or in subsequent legal discussions, can be damaging to both the investigation and the forward criminal justice process (Criminal Justice Joint Inspectorate 2014). In addition, inaccurately recording, or omitting, information from an account can lead to a loss of potentially important investigative leads (Gregory et al. 2011).

To evaluate the accuracy and completeness of interviewers' memory of events described by witnesses, research has examined the nature and accuracy of recorded details when interviewers take notes during interviews. For example, Cauchi and Powell (2009) found that notes made during interviews with child witnesses were not always accurate; 15% of notes contained at least one or more errors of commission (i.e., the inclusion of incorrect information). An evaluation of interviewers' reports of the information gleaned from adult witnesses following cognitive or structured interviews uncovered 81% and 78% accuracy respectively (Kohnken, Thurer, and Zoberbier 1994). Warren and Woodall (1999) found that interviewers' reports were 98% accurate but only accounted for, on average, 68% of the information provided by a witness. These studies indicate that information provided by witnesses is not always accurately or fully recorded in interviewers' notes. However, in such cases, it is not clear whether information was originally encoded by interviewers, but not reported, or whether interviewers did not encode the information reported by witnesses in the first place.

In addition to accurately recalling the information provided by a single witness, when conducting investigations with multiple witnesses, interviewers must also accurately monitor the source of discrete details. This is important to enable differentiation between witnesses' accounts and to assess what information is novel and what has been corroborated by others. Monitoring the source of remembered information is important in many everyday situations and enables judgements to be made about that information (Johnson, Hashtroudi, and Lindsay 1993). For example, when a person witnesses an event with at least two people involved they rely on source memory to recollect who performed what action (Kleider et al. 2008). Errors, or disruptions, in monitoring the source of information can have implications for memory, and knowledge, of an event (Johnson, Hashtroudi, and Lindsay 1993).

The Source Monitoring Framework conceptualises how people distinguish memories from different sources (Lindsay 2007) and is used to explore the mechanisms by which memories are attributed to particular events or origins (Johnson, Hashtroudi, and Lindsay 1993). Research on the source monitoring framework shows that accurately identifying the source of information depends on the quality and characteristics of the activated memory (Mitchell and Johnson 2000). For example, in eyewitness identification studies it has been argued that, when making an identification, a witness must differentiate between their thoughts and feelings when encoding an event/crime, from other features, such as familiarity with one of the line‐up members (Lindsay 2007). The mental reinstatement of these representations of memory can range from general feelings of familiarity to memory for specific features (Evans and Wilding 2012). The subjective awareness of the memory for an event is, therefore, central to understanding the source monitoring framework (Mitchell and Johnson 2000). A subjective recollection of, or familiarity with, an event can distinguish between memories for specific events and more general memories of the event. That is, it is possible to know about something without remembering the event that provided the knowledge (Gardiner, Ramponi, and Richardson‐Klavehn 2002). ‘Remembering’, then, refers to a ‘conscious recollection’ of an event, whereas, ‘knowing’ occurs with a ‘sense of familiarity’ but without conscious recollection of the event (Tulving 1985).

Research examining the source monitoring framework in applied contexts has generally focused on the source monitoring accuracy of witnesses' recall (e.g., Lindsay 2014) and memories of repeated or multiple events as recalled by children (Poole, Dickinson, and Brubacher 2014) and adults (Willén et al. 2015). Granhag et al. (2013) identified a complex case where multiple witnesses to a crime were interviewed on multiple occasions. In such cases, interviewer source monitoring errors could create a risk of contaminating witness memory. Source monitoring is important as recognition of the source of information can lead to successful recognition of other details about an event (Reisberg 2007). Source monitoring is also required when interviewing multiple witnesses, for example, if a witness provides information during an interview, the interviewer must consider the accuracy and source of any previous information provided about the same event. Memory errors, for example, misidentifying the source of specific information, may occur when interviewers consider information from multiple sources (Hanway and Akehurst 2018).

Accurately monitoring the source of information when conducting multiple interviews is challenging when combined with the inherent cognitive demands of interviewing. Cognitive load is an indicator of working memory use and the demands placed on cognitive resources when carrying out multiple and competing tasks (Dias et al. 2018; Engstrom, Landtblom, and Karlsson 2013; Van Acker et al. 2018). In the course of an investigative interview, interviewers are required to hold information provided by witnesses in memory, at the same time as assessing that information, thinking of questions to ask, and identifying the correct order in which to ask those questions. Interviewers must also keep in mind the best way to ask a question using non‐leading, open‐ended questions as much as possible (Hanway and Akehurst 2018). An increase in cognitive demands can negatively impact recall of information and increase perceived cognitive load when completing interviewing tasks (Hanway et al. 2021).

Capacity limitations of working memory can lead to cognitive load where received sensory information is not rehearsed and the processing of the information may then be restricted (Van Merriënboer and Sweller 2010). For example, interviewers may neglect to listen carefully to a witness because they are thinking about the next question they should ask (Hanway and Akehurst 2018). Also, dividing attention between two sources can increase cognitive load and impact understanding and retention (Sweller 2011). Divided attention at encoding can result in deficits in item memory, as well as in source memory (Greene, Martin, and Naveh‐Benjamin 2021). This divided attention may then increase source monitoring errors if useful source specific information (e.g., about the interviewee) is not linked with the memory of a piece of information provided by that interviewee (Mitchell and Johnson 2000). Cognitive load that results from attending to multiple cognitive tasks during an interview may, therefore, increase errors in memory recall and source monitoring for interviewers.

The current research examined the effects of increasing cognitive demands on participants' perceived cognitive load, the accuracy of their memory for information provided by multiple witnesses, their accuracy in identifying the source of information and participants' subjective experience of their memory for information and monitoring of the source of information. It was predicted that in a high cognitive load condition participants would report increased levels of perceived cognitive load, have lower accuracy scores for memory of witnesses' accounts, and lower source monitoring accuracy, when compared with those in a no additional cognitive load condition (Hypothesis 1). For interviewers' subjective experiences, it was predicted that in the high cognitive load condition, participants would report a lower proportion of ‘remember’ and ‘know’ responses, and a higher proportion of ‘guess’ responses, when compared with those in the no cognitive load condition (Hypothesis 2).

## 1 Method

### 1.1 Design

For this preregistered (see Supporting Information) independent‐groups study, there was one between‐subjects factor, cognitive load, with two levels: high cognitive load (HCL) and no additional cognitive load (NCL; control). The dependent variables were perceived cognitive load (PCL), memory accuracy and Source Monitoring (SM) accuracy. Participants' subjective experiences of (i) recognising information and (ii) recognising the source of the information were also measured. The tasks for each condition were designed to replicate some cognitive demands that have been identified as inherent in investigative interviewing (i.e., to listen to the witness, remember information, judge information and think of questions to ask; Fisher et al. 2014; Hanway and Akehurst 2018).

### 1.2 Participants

A priori G*power analysis for a one‐way *t*‐test, with two independent groups based on an alpha level of 0.05 and a medium effect size of 0.50, indicated that a sample size of 102 participants was required for this study to have sufficient power at 0.80 (Faul et al. 2009). The sample of 102 participants comprised 65 females and 37 males, who were aged 18–45 years (*M*_age_ = 21.02 years, SD = 4.38 years). 91% of the sample reported their nationality as British. Of the 102 participants, 99 reported English as their first language and the other three reported English as their primary language. The participants, who were university staff and students, were recruited via a local participant pool and advertisements placed in university buildings. First year undergraduate psychology students were offered course credit for their participation. Other participants were offered a monetary incentive of £5 for taking part. All participants were informed that they would be required to attend for one 45‐min session during which they would take the role of a police interviewer investigating a crime that had been witnessed by several people.

### 1.3 Materials

#### 1.3.1 Stimulus Interviews

Five student actors served as mock‐witnesses to a fictitious crime event. The mock‐witnesses were all females of similar age, but with different hair styles and wearing different clothing. They were each given a script to learn relating to the mock‐crime which involved a man attacking a woman in a nightclub. Each of the mock‐witnesses acted as if they were present in the nightclub where the attack took place. They were asked to describe the event as naturally as possible, including a number of unique and generic details provided in their script. To prevent order effects, each mock‐witness described the crime event in a different order (e.g., one witness described the man and then the assault and another described details of the assault and then of the man). The mock‐witnesses were each interviewed separately about the crime event and their interviews were audio and video recorded.

During their interviews, the witnesses were asked the same open question and they each provided a free recall narrative containing four unique details and 16 generic details of the crime. That is, 20 details in total were mentioned by each of the five witnesses but each witness provided four unique details. For example, one mock‐witness provided four unique details about the incident (i.e., they went to ‘Clouds’ nightclub, she was with ‘four’ friends, she was on the dance floor with ‘Chloe’, and the man punched the woman in the ‘face’). The four other mock‐witnesses provided generic information about these unique details (i.e., they went to a nightclub, she was with her friends, she was on the dancefloor with a friend, and the man punched the woman).

Each of the five interviews lasted for approximately two minutes (*M* = 2.08 min, SD = 15.38 s). The five interviews, presented in the same order, were combined into one media file. Each interview was separated by a screen with the witness number, one through to five, introducing each witness. The break between interviews lasted for 5 s. The media file, which lasted for 11 min 12 s, was played in totality as stimulus material for each participant.

#### 1.3.2 Perceived Cognitive Load (PCL) Measure

To measure participants' perceived cognitive load, the National Aeronautics and Space Administration, Task Load Index (NASA‐TLX) was used. This questionnaire combines information about the magnitude and source of six related factors to derive a sensitive and reliable estimate of workload (Hart and Staveland 1988). The NASA‐TLX was designed to be used during, or immediately after, a task. It has been widely used in a variety of settings (e.g., air traffic control and medical training) to measure the cognitive load perceived by participants when they have completed a task (Hart 2006; Rizzo et al. 2016).

The NASA‐TLX multi‐dimensional rating scale questionnaire evaluates participants' subjective workload ratings. The scale items comprise mental demand, physical demand, temporal demand, performance, effort and frustration (Hart 2006). Each item is measured on a 20‐point scale from low to high (except for performance which is measured on a scale from good to poor). A weighted score is obtained by completing 15 pairwise comparisons of the six scale items. For each pair of scale items, one item is selected that the participant feels is more relevant for them when completing the task (Hart and Staveland 1988). For this study and following the scoring procedure devised by Hart and Staveland (1988), an overall PCL score out of 100 was calculated. Each scale item score (rating score) was multiplied by the number of times that item was selected in the pairwise comparisons (giving an adjusted score); the six adjusted item scores were then totalled and divided by 15 to obtain the overall PCL score which could range from 0 to 100.

#### 1.3.3 Memory and Source Monitoring Task

Participants were presented with 20 questions, in a random order, about the 20 unique details provided by the five mock‐witnesses. They were given multiple‐choice answers (four choices) with just one correct answer for each question. For example, one question was ‘what colour jacket was the suspect wearing?’ The answer options were Red/Blue/Black/White and the correct answer was ‘black’.

For the SM task, participants were presented with their answer to each of the 20 multiple‐choice questions (e.g., previously you were asked the question ‘How many friends did the witness say she went out with?’ The answer you gave to this question was ‘four’) they were then asked to indicate which witness provided the information (i.e., witness 1, 2, 3, 4, or 5). As each mock witness had given a unique set of details during their interview, there was only one source, (i.e., correct answer) for each unique detail.

#### 1.3.4 Subjective Experiences of Remembering and Source Monitoring

Participants' subjective experiences during the memory task and SM task were measured as the proportions of remember, know, or guess (R/K/G) responses they gave relating to their answers to the 20 unique detail questions and the 20 SM questions. The two forms of recognition memory, recollection (remember) and familiarity (know) were included as response options to distinguish between memories for specific details and more general memories of the details. ‘Guess’ was added as a response criterion as, when measuring participants' subjective awareness, ‘guess’ can prevent ‘know’ being selected when participants actually guessed the answer (Gardiner, Ramponi, and Richardson‐Klavehn 2002). Including ‘guess’ also helped to confine R–K judgements to confidently recognised items, which can encourage more accurate ‘know’ responses (Eldridge, Sarfatti, and Knowlton 2002). Instructions for completing the R/G/K task were drawn from the definitions outlined by (Williams and Lindsay 2019); see Supporting Information.

### 1.4 Procedure

On arrival at the lab, all participants were required to read an information sheet and gave written informed consent. To ensure equal numbers (*N* = 51) in each of two conditions, participants were pseudo‐randomly allocated to one of the two conditions, high cognitive load (HCL) or no additional cognitive load (NCL). All participants were instructed to take the role of a police interviewer and were told that their task was to investigate a reported crime of assault on a 23‐year‐old woman. Participants were informed that five witnesses to the crime had been interviewed regarding the incident, the interviews had been recorded and they would view the recorded witness interviews.

Participants were asked to watch the interviews carefully as if they were the interviewer and listen to everything the witnesses said. They were also informed that they would be asked some questions after they watched the interviews. Participants in the NCL condition were asked to consider carefully what the witnesses said so that they could clearly understand the witnesses' knowledge of the event. In the HCL condition, participants were additionally instructed that whilst watching the interviews, they should consider carefully what the witnesses said so that they could clearly understand the witnesses' knowledge of the event. They were also informed that an additional task was to identify follow‐up questions to ask each witness once they had given their accounts. Hence, whilst listening to each of the witnesses, participants in the HCL condition were instructed to think about what further information they would like to obtain from the witnesses to help the investigation and how they should word their questions to obtain that information. To ensure compliance with this aspect of their task, participants in the HCL condition were provided with a pen and paper and were asked to write their questions down whilst each interview was in progress.

After receiving instructions specific to their experimental condition, all participants were asked to wear headphones to reduce distractions and they watched the five recorded interviews on a computer screen. Immediately after watching all five of the interviews, all participants completed the PCL measure using the NASA‐TLX scale, which was presented to participants via a tablet application. They then carried out a 5‐min distraction task that required them to work through some unrelated number puzzles.

Following the distraction task, all participants were asked 20 multiple‐choice questions about the 20 details that had been provided in the accounts given by the witnesses. The questions were presented on a computer using Qualtrics survey software and were presented in random, differing orders for each participant. For each question, participants were provided with four answer choices and they were able to select just one answer. Once participants had selected their answer, they were asked to provide their subjective experience of their memory of the information (i.e., did they R/K/G their answer). For clarity, the definitions of R/K/G were presented to participants on a laminated card and they were able to refer to the definitions throughout the time they were answering the R/K/G questions.

Participants were then presented with the answers they had given to the 20 multiple‐choice questions in turn and were asked SM questions for each of the 20 answers they had provided. They were asked which of the five witnesses had provided the information (i.e., witness 1–5) relating to each multiple‐choice question. When completing this section, a picture of each witness (1–5) was provided to participants on a laminated sheet that they could refer to when selecting their responses. After each SM question was answered, participants were asked to provide their subjective experience of their SM memory for the related information (i.e., did they R/K/G their answer).

Finally, participants were asked to rate for each witness (1–5), using 7‐point scales, ‘I am confident in the accuracy of my memory for information given by witness (1–5)’ from [1] strongly disagree to [7] strongly agree; ‘I was motivated to remember the content of the account given by witness (1–5)’, from [1] strongly disagree to [7] strongly agree; ‘I found remembering the content of the witness’ account (1–5)’, from [1] extremely easy to [7] extremely difficult. Participants in the HCL condition were also asked ‘I was motivated to think about questions whilst I was listening to the account of witness (1–5)’, from [1] strongly disagree to [7] strongly agree.

As a manipulation check, participants were asked to write down the instructions they were given by the researcher at the outset of the session before they watched the witnesses' accounts. They were also asked if they personally knew any of the five mock‐witnesses. Demographic details including age, gender, nationality, ethnicity and first language, were also recorded. On completion, a verbal debrief was provided for all participants and they were thanked for their time and effort. Participation in the study lasted approximately 40 min.

## 2 Results

### 2.1 Manipulation Check

All 102 participants passed the manipulation check and accurately reported their instructions. As per their instructions, participants in the NCL condition confirmed they were required to watch the interviews carefully and participants in the HCL condition confirmed that they were asked to watch the interviews carefully and to think of questions to ask the witnesses. One participant in the NCL condition indicated that she knew one of the witnesses. Analyses were conducted with and without this participant's data, which revealed no differences in the results. For completeness, the data of all 102 participants were included in the analyses and are reported in the results.

### 2.2 Hypothesis Testing

#### 2.2.1 Perceived Cognitive Load (PCL)

A *t*‐test was conducted to examine differences between conditions in PCL scores. As predicted, participants in the HCL condition scored higher on the PCL measure (*M* = 57.63, SD = 11.41) than those in the NCL condition (*M* = 50.59, SD = 12.15), *t* (100) = 3.02, *p* = 0.003, 95% CI [2.41, 11.67], *d* = 0.60. That is, participants in the HCL condition who were required to think of questions, and write them down, whilst watching the witness interviewers reported higher perceived cognitive load than did those in the NCL condition who merely watched the witnesses' interviews.

#### 2.2.2 Memory Accuracy for Unique Details

Accuracy scores for recognition of unique details of the witnesses' accounts were calculated as the percentage of correct responses to the 20 multiple‐choice questions. A *t*‐test was conducted to examine differences between conditions for percentage accuracy of recognised information. As predicted, there was a difference in percentage accuracy between the two conditions. Percentage accuracy for unique details was lower in the HCL condition (*M* = 77.16, SD = 11.80) than in the NCL condition (*M* = 83.24, SD = 9.63), *t* (100) 2.85, *p* = 0.005, 95% CI [1.85, 10.31], *d* = 0.57.

#### 2.2.3 Accuracy of Source Monitoring

First, the percentage accuracy scores for monitoring the source of the unique details were calculated as the percentage of correct responses to the 20 SM questions, irrespective of whether the answer provided for the unique detail had been correct (i.e., monitoring the source of information without considering the accuracy of unique detail questions). A *t*‐test was conducted to examine differences between conditions for percentage accuracy of SM. The prediction that there would be a difference in SM scores was not supported. There was no significant difference in accuracy of SM between participants in the HCL condition (*M* = 39.71, SD = 12.90) and those in the NCL condition (*M* = 43.04, SD = 12.33), *t* (100) = −1.33, *p* = 0.185, 95% CI [−8.29, 1.63], *d* = 0.27.

Second, percentage accuracy scores were then calculated for the SM questions following a correct answer to the unique detail questions (i.e., recognition of both the unique detail and the source of the detail was correct). A *t*‐test was conducted to examine differences between conditions for percentage accuracy of SM for correct details. There was no significant difference in accuracy of SM between participants in the HCL condition (*M* = 34.41, SD = 12.56) and those in the NCL condition (*M* = 38.14, SD = 12.33), *t* (100) = 1.44, *p* = 0.154, 95% CI [−1.42, 8.87], *d* = 0.30.

#### 2.2.4 Subjective Experiences of Remembering

Participants' subjective experience of their memory for unique details and SM were measured as the proportions of remember, know, or guess (R/K/G) responses they gave relating to their answers to the 20 memory questions and the 20 SM questions.

Memory of unique details. Pearson's correlations indicated that there were significant, but moderate, associations between the proportion of R/K/G responses related to participants' subjective experience of their answers to the unique detail questions (remember and know, *r* (102) = −0.78, *p* < 0.001; remember and guess *r* (102) = −0.65, *p* < 0.001). There was no correlation between know and guess responses, *r* (102) = 0.03, *p* = 0.760. As a result, the assumption of an absence of multicollinearity was met and to reduce Type 1 error, a one‐way between‐groups MANOVA with Condition (HCL vs. NCL) as the only factor was conducted. The proportion of remember, know and guess responses relating to the unique detail questions were the three dependent variables. Separate MANOVAs were conducted for all answers, correct answers and incorrect answers, that participants provided to the unique detail questions.

For all answers to subjective experience of the unique detail answers, the MANOVA revealed a significant multivariate main effect for condition, Wilks Λ = 0.86, *F* (3, 98) = 7.93, *p* = 0.001, *η* ^2^_p_ = 0.14. As predicted, the univariate main effects revealed a difference in the proportion of remember and guess responses between HCL and NCL conditions. Those in the HCL condition reported ‘remembering’ fewer of their answers to the unique detail questions and ‘guessing’ more of their answers than did those in the NCL condition. Contrary to the prediction, there was no significant difference between conditions for the reported subjective experience ‘know’ (see Table 1).

**TABLE 1:** Mean and standard deviation scores for R/K/G including results for all answers, correct answers and incorrect answers to unique detail questions for each condition.

Answers	R/K/G	HCL		NCL				
		Mean	SD	Mean	SD	*t* (1,100)	*p*	*d*
All answers	Remember	0.50	0.16	0.60	0.17	9.66	0.002	0.61
	Know	0.25	0.11	0.23	0.15	0.81	0.370	0.15
	Guess	0.25	0.11	0.17	0.10	15.19	< 0.001	0.77
Correct answers	Remember	0.62	0.17	0.71	0.19	2.38	0.019	0.50
	Know	0.25	0.14	0.22	0.17	−1.07	0.289	0.19
	Guess	0.13	0.09	0.08	0.07	−3.22	0.002	0.63
Incorrect answers	Remember	0.07	0.13	0.03	0.12	−1.57	0.120	0.32
	Know	0.24	0.23	0.27	0.32	0.65	0.517	0.11
	Guess	0.67	0.29	0.66	0.34	−0.27	0.791	0.03

To examine any differences between conditions for R/K/G responses, that may have been dependent on the accuracy of recognition of details, analyses were conducted for correct and incorrect answers to recognition of the unique details. For correct answers relating to unique details, the MANOVA revealed no significant multivariate main effect for condition, Wilks Λ = 0.95, *F* (3, 98) = 1.84, *p* = 0.146, *η* ^2^_p_ = 0.05. For incorrect answers to the unique detail questions, the MANOVA also revealed no significant multivariate main effect for condition, Wilks Λ = 0.99, *F* (3, 98) = 0.40, *p* = 0.754, *η* ^2^_p_ = 0.01 (see Table 1).

Subjective experience of remembering source information. For subjective experience of recognition of the source of information, Pearson's correlations indicated that there were significant, but moderate, associations between the proportions of remember, know and guess responses out of 20: for remember and know, *r* (102) = −0.49, *p* < 0.001; for remember and guess *r* (102) = −0.59, *p* < 0.001; and for know and guess, *r* (102) = 0.41, *p* < 0.001. As a result, the assumption of an absence of multicollinearity was met and to reduce Type 1 error, a one‐way between‐groups MANOVA with Condition (HCL vs. NCL) as the only factor was conducted. Remember, know and guess in relation to responses to SM questions were the three dependent variables.

There was no significant multivariate main effect for Condition, Wilks Λ = 0.96, *F* (2, 99) = 2.35, *p* = 0.101, *η* ^2^_p_ = 0.05. The univariate main effects revealed a difference in the proportion of guess responses for SM questions between the HCL and NCL conditions. Those in the HCL condition reported ‘guessing’ more answers to the SM questions than did those in the NCL condition, there was no significant difference between conditions for the reported subjective experiences of ‘remember’ and ‘know’.

To examine any differences between conditions for R/K/G responses to SM questions, dependent on the accuracy of responses, additional exploratory analyses were conducted. For correct SM responses, the MANOVA revealed a significant multivariate main effect for condition, Wilks Λ = 0.91, *F* (3, 98) = 3.23, *p* = 0.026, *η* ^2^_p_ = 0.09. For incorrect SM responses, the MANOVA revealed there was no significant multivariate main effect for condition, Wilks Λ = 0.97, *F* (3, 98) = 1.13, *p* = 0.342, *η* ^2^_p_ = 0.03. (see Table 2).

**TABLE 2:** Mean and standard deviation scores for R/K/G responses including results for all SM responses, and responses following correct and incorrect answers, for each condition.

Answers	R/K/G	HCL		NCL				
		Mean	SD	Mean	SD	*t* (1,100)	*p*	*d*
All answers	Remember	0.25	0.17	0.29	0.18	1.30	0.257	0.23
	Know	0.33	0.15	0.37	0.16	1.06	0.305	0.26
	Guess	0.41	0.16	0.34	0.17	4.72	0.032	0.43
Correct answers	Remember	0.37	0.23	0.40	0.22	0.70	0.485	0.13
	Know	0.33	0.15	0.37	0.16	1.06	0.305	0.26
	Guess	0.29	0.21	0.21	0.15	−2.17	0.033	0.44
Incorrect answers	Remember	0.18	0.18	0.20	0.19	0.50	0.615	0.11
	Know	0.34	0.17	0.36	0.21	0.66	0.514	0.11
	Guess	0.48	0.20	0.44	0.22	−1.06	0.291	0.19

### 2.3 Confidence, Ease of Remembering and Motivation

In the post recall questionnaire, the dependent variables were confidence, ease of remembering the witnesses' accounts, motivation to remember the accounts, and for the HCL condition, motivation to think of questions to ask the witnesses. As participants' scores for each witness (1–5) contributed the same weight to each of the dependent variables, a composite score for each variable was calculated as the mean of participants' scores for each witness (1–5) for each variable. A series of Pearson's correlations were conducted to determine whether the dependent variable composite scores were correlated with each other. Only two of the variables were moderately correlated, therefore, there was an absence of multicollinearity. A series of *t*‐tests were conducted for each dependent variable with condition (HCL vs. NCL) as the only factor. To reduce the risk of Type 1 errors a Bonferroni adjustment was made (i.e., the alpha level of 0.05 was divided by the number of tests to be performed [4] to give an alpha of 0.013). Differences between participants' motivation, confidence and their ease of remembering the account for each witness were investigated.

For participants' confidence in the accuracy of their memories for witnesses' accounts, there was no difference between the HCL (*M* = 4.47 SD = 0.91) and NCL (*M* = 4.69 SD = 1.11) conditions, *t* (100) = −1.09, *p* = 0.278, 95% CI [−0.62, 0.18], *d* = 0.22. For participants' ratings of ease of remembering witnesses' accounts, participants in the HCL condition rated remembering the accounts as more difficult than did those in the NCL condition, HCL *M* = 3.93 (SD = 1.17), NCL *M* = 3.38 (SD = 1.00), *t* (100) = 2.59, *p* = 0.011, 95% CI [1.22, 0.98], *d* = 0.51, where ratings of 1 = extremely easy and ratings of 7 = extremely difficult. For participants' motivation to remember the witnesses' accounts, participants in the HCL condition rated their motivation to remember the accounts as higher than did those in the NCL condition, HCL (*M* = 5.05 SD = 0.95), NCL (*M* = 5.51 SD = 0.87), *t* (100) = −2.53, *p* = 0.013, 95% CI [−0.82, −0.10], *d* = 0.51, where 1 = strongly disagree and 7 = strongly agree. Participants in the HCL condition were also asked to rate their motivation to think of questions to ask whilst listening to the witnesses' accounts, *M* = 5.56 SD = 1.00, where 1 = strongly disagree and 7 = strongly agree.

## 3 Discussion

The current findings indicate that participants' perceived a higher cognitive load when they were required to complete additional cognitive tasks, compared with simply watching and remembering the content of witnesses' accounts. As predicted, participants who were asked to think about follow‐up questions, and write them down, were less accurate in terms of their memory of unique details given by each witness than those who were not asked to think about questions. Contrary to our predictions, however, there were no differences between conditions in SM accuracy.

For participants' subjective ratings of their memory for unique details, participants in the HCL condition reported a lower proportion of ‘remember’ responses and a higher proportion of ‘guess’ responses, when compared with those in the NCL condition. When considering participants' subjective experience of SM, those in the HCL condition reported a higher proportion of ‘guess’ responses for their subjective experience of SM, when compared with those in the NCL condition. There were no differences in ‘remember’ and ‘know’ responses across conditions.

To test participants' memory of the witnesses' accounts, the current research used a recognition rather than a recall task. Despite the fact that recognition memory is generally more accurate and less effortful than recall (Yonelinas 2002), the current pattern of impaired recognition memory accuracy for unique details in witness accounts when under high cognitive load broadly replicates previous research showing impaired performance by mock‐interviewers on recall tasks when under high cognitive load (Hanway et al. 2021). If interviewers cannot recognise information because it has not been encoded or it is not available for retrieval, then the amount and accuracy of information recalled by interviewers will inevitably be reduced (e.g., Cauchi and Powell 2009; Lamb et al. 2000; Warren and Woodall 1999).

Interviewers attend to information provided by a witness and consider whether that, or similar, information has been previously given. If information has been provided by another witness, interviewers must then decide who provided the information. Some SM decisions are rapid and automatic requiring less conscious thought, however, other decisions are more effortful and require conscious decision making (Johnson, Hashtroudi, and Lindsay 1993). Yonelinas (2002) suggested that remembering is more likely to be negatively affected by divided attention than perceived familiarity of information (knowing). In the current study, participants who were required to complete additional cognitive tasks remembered fewer unique details than did those who merely watched the witnesses' interviews, however, there were no differences in know responses across cognitive load conditions. Indicating that remembering was impacted by divided attention but knowing was not.

In applied settings, interviewers described that when dealing with complex crimes, they are often required to interview multiple witnesses about the same crime or the same offender (Hanway and Akehurst 2018). In the current study, no difference in accuracy for monitoring the source of information between participants in the HCL and NCL conditions was found. However, it is worthy of note that all participants performed poorly on the SM task (i.e., SM accuracy was only 34% for the HCL condition and 38% for the NCL condition). The low SM accuracy scores suggest that, during divided attention tasks, such as interviewing, useful source specific information may not be linked to the details of the information, which can increase SM errors (Mitchell and Johnson 2000).

The low SM accuracy rates mirror those of previous studies, which suggest people are more likely to confuse memories from similar sources (Lindsay, Johnson, and Kwon 1991). For the current study, the sources of information (i.e., the five witnesses) were similar, in that, they were of the same ethnicity, age and gender. The five witnesses also provided similar details that differed in specificity. That is, one witness provided a unique detail (e.g., ‘we went to ‘Clouds’ nightclub’), whereas the four other witnesses provided generic information (e.g., ‘we went to a nightclub’). Thus, low SM rates may also be due to the nature of the information (i.e., generic or specific) that was provided by the five witnesses.

Although participants in the HCL condition wrote down their follow‐up questions, no participants made notes of what was said by the witnesses. Actively attending to aspects of source information during an event (e.g., by noting down information) has been shown to enhance source memory (Lindsay 2007). However, as note taking can be cognitively demanding (e.g., Piolat, Olive, and Kellogg 2005) and may divide attention, the aim of the current study was to focus participants' attention on the cognitive tasks of thinking about questions and remembering information. It is acknowledged that, in operational settings, interviewers may take notes during interviews and may be available during subsequent interviews to aid interviewers recall. However, this is not always the case, for example in intelligence gathering contexts, when interviewing at the scene of an incident, or when interviewing child witnesses. Future research should examine whether note taking has an impact on interviewers' PCL or their accuracy for memory for unique details and the source of information.

The nature of the tasks that were completed for the current study (i.e., passively watching the account of an interviewee and a recognition memory task) are not akin to those experienced by investigators in the field. For example, interviewers in practise would engage with interviewees, through questioning, during their interviews. To increase the generalizability of the current findings it is recommended that future research examine whether the current results replicate with trained investigative interviewers in applied settings.

The current research has highlighted that keeping track of information provided by multiple witnesses when they give their account of a crime is challenging for interviewers. This challenge is reflected in impaired memory accuracy and impaired ability to monitor the source of specific information. To reduce such errors, interviewers should be trained to not only carefully attend to information provided by witnesses, but also to attend to the source of information. This may assist interviewers' later recall of key information when interviewing additional witnesses to the same event. Meanwhile, future research should focus on the development of assistive strategies, tools or techniques to support interviewer cognition.

## Author Contributions

The first and second authors conceived the research idea. The first and second authors designed the research with feedback and reviews from the third and fourth authors. The first author conducted the research, analysed and interpreted the data, and wrote the research paper. The second, third, and fourth authors provided feedback on the research paper.

## Conflicts of Interest

The authors declare no conflicts of interest.

## Supporting information


Data S1.

Data S2.

## Data Availability

The data that support the findings of this study are available from the corresponding author upon reasonable request.
