# Supplementary material for: Who Said What? The Effects of Cognitive Load on Source Monitoring and Memory for Multiple witnesses' Accounts
Source: Appl Cogn Psychol. 2024 Nov 27;38(6):e70011. doi: 10.1002/acp.70011 (PMC11602681; doi:10.1002/acp.70011)
Supplement: Supplementary file 1 — Data S1. [file ACP-38-e70011-s001.docx]

## **Supplementary materials**

Instructions for completing the R/G/K task were drawn from the definitions outlined by Williams and Lindsay (2019). The instructions given were:

REMEMBER. You have an experience of recollection for the exact answer. This could include being consciously aware of some aspect or aspects of what was experienced at the time the answer was given in the interviews phase (e.g., aspects of the physical appearance of the witness, or of something that happened, or of what you were thinking or doing at the time). In other words, you should choose “Remember” if you have a sense of yourself in the past and/or the question brings back to mind a particular association, image, or thought, from the time of interviews. *For example, if you see someone on the street, you may think, “Who is that? Oh yes, it’s the person I saw in the queue in the book shop. I remember thinking what a funny hat they had on…”*

KNOW. You feel that you just know that the answer was a detail you heard in the interviews phase, but you cannot consciously recollect anything about its actual occurrence or what was experienced at the time of its occurrence. In other words, you should choose “Know” if you know the answer was a detail you were given, but you cannot recollect any details associated with seeing or hearing it. *For example, if you see someone on the street, you may think “Who is that? I know I've seen that person before, but I don't recall where that would have been…”*

GUESS. You do not have any memories or feelings associated with the question, and you are simply guessing that the answer was one of the details given in the interviews phase.
