# Supplementary material for: Who Said What? The Effects of Cognitive Load on Source Monitoring and Memory for Multiple witnesses' Accounts
Source: Appl Cogn Psychol. 2024 Nov 27;38(6):e70011. doi: 10.1002/acp.70011 (PMC11602681; doi:10.1002/acp.70011)
Supplement: Supplementary file 2 — Data S2. [file ACP-38-e70011-s002.docx]

1) Have any data been collected for this study already?

No, no data have been collected for this study yet.

2) What's the main question being asked or hypothesis being tested in this study?

The main goal of this research is to examine the effects of increased cognitive demands on

investigative interviewers’ perceived cognitive load, their recall of information provided in

the accounts of multiple witnesses, and their source monitoring errors. Specifically, we will

examine the following questions: What are the effects of increased cognitive demands on the

interviewers’, i) perceived cognitive load, ii) accuracy of recall of multiple witnesses’ accounts,

iii) subjective experience of recalling information from multiple witnesses, indicated by

remember (recollection), know (familiar), or guess, responses, iv) source monitoring errors

when identifying which witness provided the information, and v) subjective experience of

recalling which witness provided the information, indicated by remember (recollection),

know (familiar), or guess, responses?

We predict that, in a high cognitive load (HCL) condition, participants, who will be asked to

formulate questions in their heads whilst watching multiple interviews, will report increased

levels of cognitive load when compared with those in a no cognitive load (NCL) condition,

who will be given instructions to merely watch and listen to the accounts. We also predict

that participants in the HCL condition will have lower accuracy scores, for their recall of

details about the witnesses’ accounts, when compared with those in the NCL condition. For

interviewers’ subjective experience of recall, we predict that in the HCL condition,

participants will report i) a lower proportion of remember responses, ii) a lower proportion of

know responses, and iii) a higher proportion of guess responses, when compared with those in

the NCL condition. Further, participants in the HCL condition will make an increased number

of source monitoring errors compared with those in the NCL condition. For interviewers’

subjective experience of source monitoring, participants in the HCL condition will also report

i) a lower proportion of remember responses, ii) a lower proportion of know responses, and iii)

a higher proportion of guess responses, when compared with those in the NCL condition.

3) Describe the key dependent variable(s) specifying how they will be measured.

The dependent variables will be:

1) Perceived cognitive load. The participants’ self-reported cognitive load when performing

the interview observation task (as measured by the NASA-TLX).

2) Accuracy of answers to 20 forced-choice recognition questions, based on information

presented in the witnesses’ accounts. Accuracy will be measured as the proportion of correct

responses to the questions.

3) Participants’ subjective experience of recall will be measured as the proportions of

remember, know, and guess, (R/K/G) responses for their answers to the 20 forced-choice

recognition questions.

4) Accuracy of source monitoring will be measured as the proportion of correct answers to 20

source of information questions.

5) Participants’ subjective experience of source monitoring will be measured as the

proportions of remember, know, guess, (R/K/G) responses for their answers to the sources of

information questions.

4) How many and which conditions will participants be assigned to?

There will be one independent variable of cognitive load, with two levels: no cognitive load

(NCL) and high cognitive load (HCL). To assign equal numbers of participants per condition,

participants will be pseudo-randomly assigned to one of the conditions.

5) Specify exactly which analyses you will conduct to examine the main question/hypothesis.

To test our hypotheses, independent t-tests will be used. The IV will be cognitive load: NCL

vs. HCL. The dependent variables will be: 1) PCL, 2) Accuracy of recall (i.e. the proportion of

accurate responses to 20 forced-choice recognition questions), 3) Subjective experience of

recall (i.e., the proportion of R/K/G responses for their 20 forced-choice recognition answers),

4) Accuracy of source monitoring (i.e. the proportion of accurate responses to the source of

information questions), and 5) Subjective experience of source monitoring (i.e. the proportion

of R/K/G responses for their source of information answers).

6) Describe exactly how outliers will be defined and handled, and your precise rule(s) for

excluding observations.

Data will be checked for missing values and outliers. Participants’ data will only be excluded if

it is clear that the data has been recorded incorrectly (e.g. equipment failure). As outliers may

affect some analyses, any data that is an extreme outlier (i.e. beyond 3 SDs from the mean)

will be removed. Descriptive statistics will be reported pre and post removal of data.

7) How many observations will be collected or what will determine sample size?

No need to justify decision, but be precise about exactly how the number will be determined.

102 participants will be recruited. G*power analysis for a one-way t-test, with two

independent groups based on alpha of 0.05, power of 0.80 and a medium effect size of .5, gives

a desired sample size of 102.

8) Anything else you would like to pre-register?

(e.g., secondary analyses, variables collected for exploratory purposes, unusual analyses planned?)

Nothing else to pre-register.
